# Supplementary figures and images for: Spatiotemporal metabolic responses to water deficit stress in distinct leaf cell-types of poplar
Source: Front Plant Sci. 2024 Mar 1;15:1346853. doi: 10.3389/fpls.2024.1346853 (PMC10940329; doi:10.3389/fpls.2024.1346853)

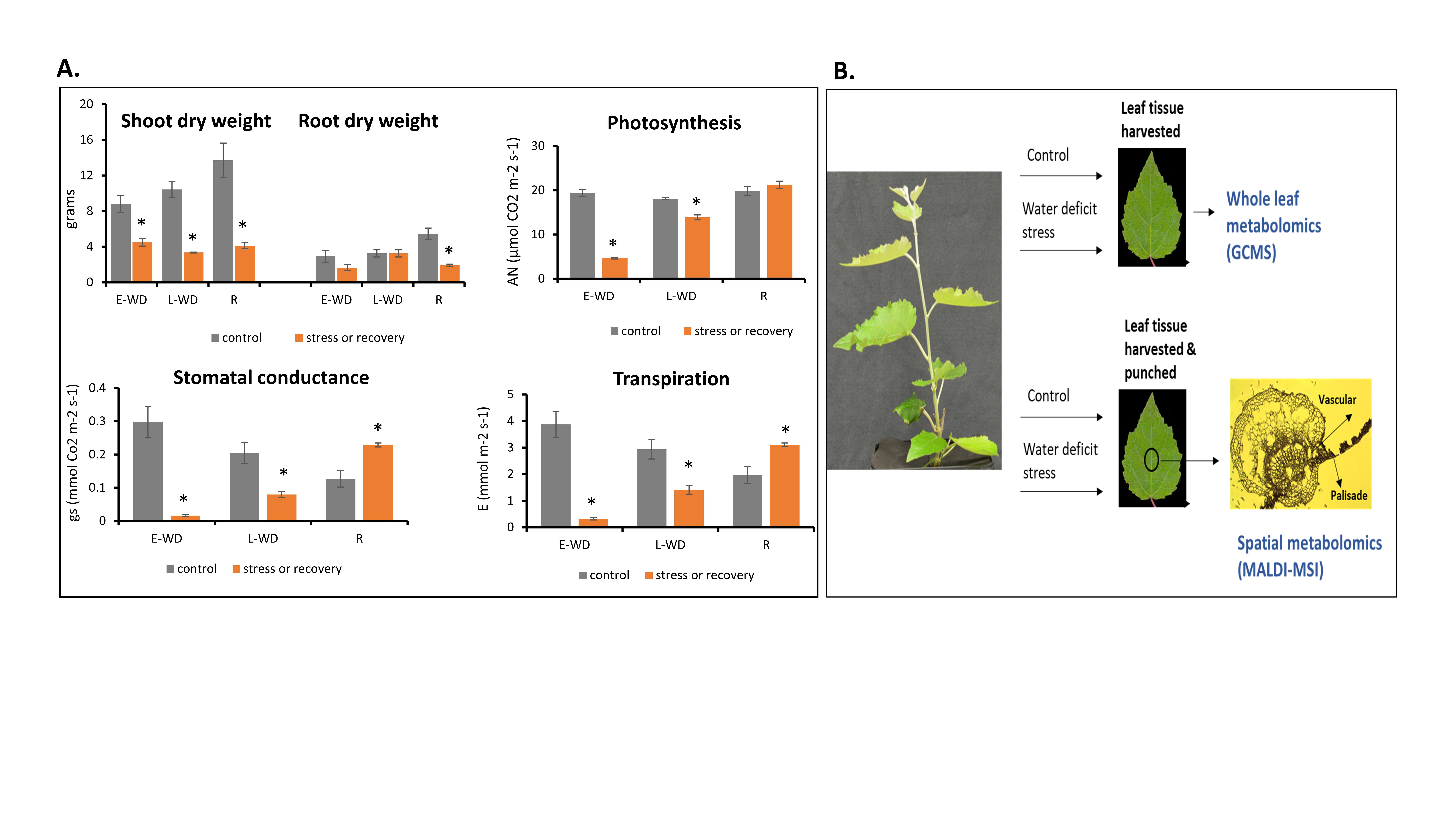

Supplement: Supplementary Figure 1 — Water deficit stress alters plant dry weight and leaf physiological parameters in poplar. (A) Shoot and root biomass and gas exchange parameters, photosynthesis (A), conductance (gs) and transpiration (E) measured from plants exposed to early water deficit (E-WD) stress (30-35% relative SWC), late water deficit (L-WD) stress (water level maintained for 10d at 30-35% relative SWC) and recovery from stress (R) and data were collected. Data averaged from four biological replicates and t-test was used for statistical analysis. *represents pvalue<0.05. (B) Leaf samples were collected for whole leaf tissue and spatial cell type specific metabolite profiling. Six biological replicates were harvested in any given condition. Matrix-assisted laser desorption Ionization-mass spectrometry imaging (MALDI-MSI) and Gas chromatography-mass spectrometry (GC-MS) analysis. [file Image_1.tif]

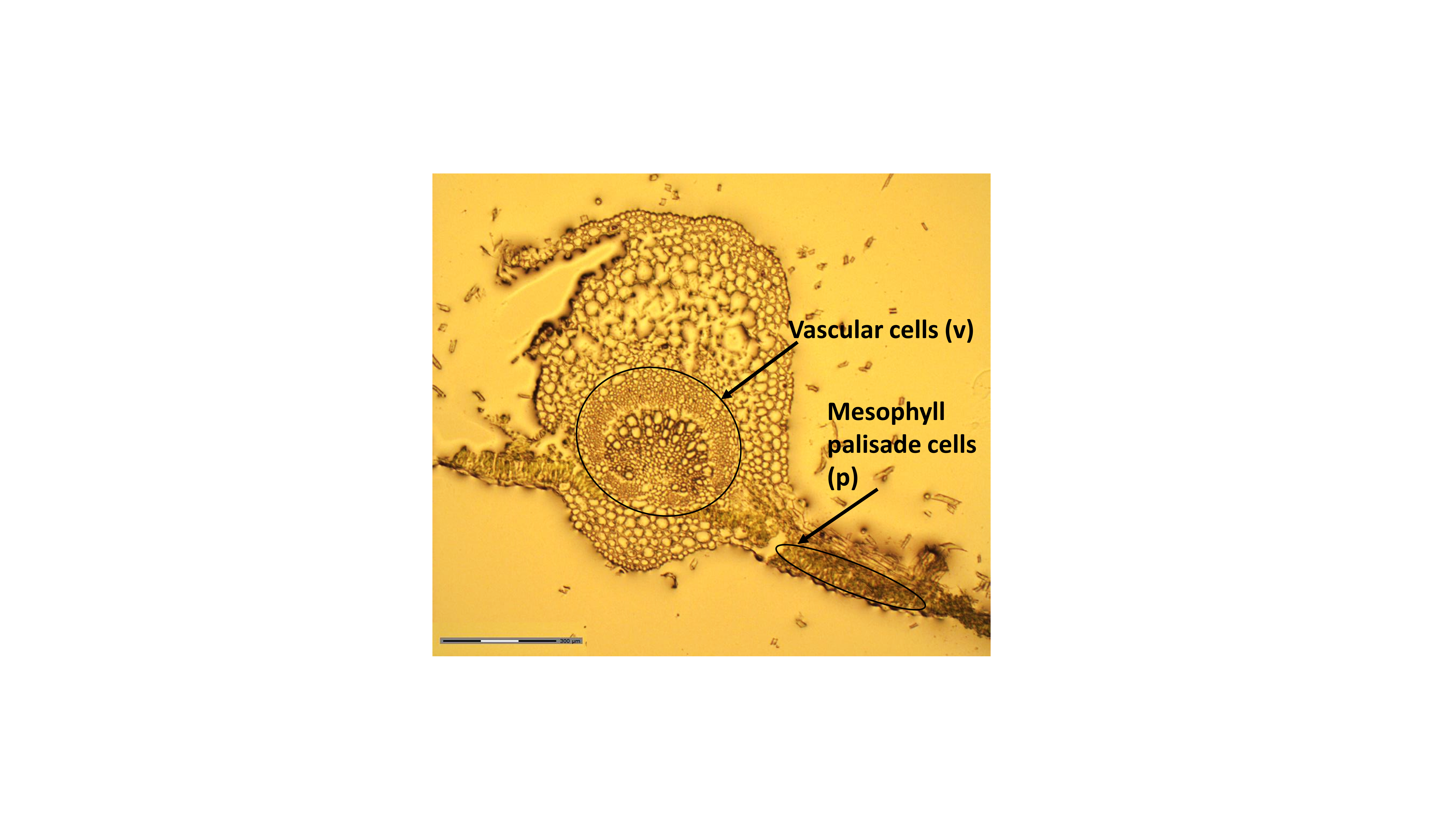

Supplement: Supplementary Figure 2 — Cryosectioning of a poplar leaf tissue. Cryosection highlighting mesophyll palisade (p) and vascular (v) cell types used for MALDI MSI to generate spatial metabolome data. Scale bar represents 300µm. [file Image_2.tif]

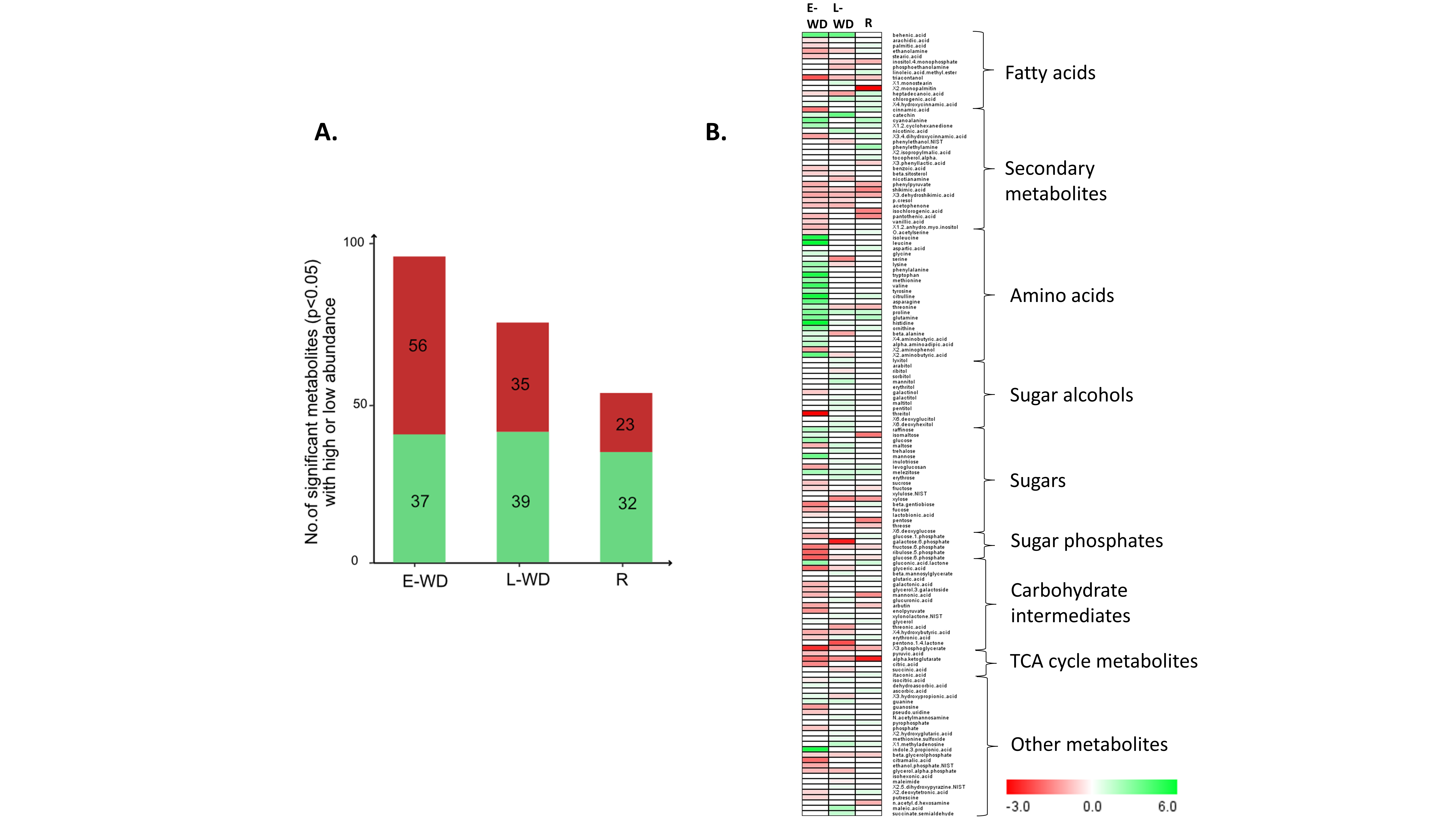

Supplement: Supplementary Figure 3 — Whole leaf tissue-based metabolomics identified water deficit stress responsive metabolites. (A) Total no. of metabolites significantly altered during early (E-WD) and late (L-WD) water deficit stress and recovery (R) conditions in whole leaf tissue of poplar. Green and red bars highlight metabolites with increased or reduced abundance levels during stress and recovery conditions. Data averaged from three biological replicates and one-way ANOVA was used for statistical analysis (p<0.05). (B) Total significant metabolites were categorized into broader metabolic classes using KEGG pathway analysis. The heatmap shows metabolite abundance levels in E-WD, L-WD and R conditions. Data averaged from 3 biological replicates and ANOVA (p<0.05) was used for statistical analysis. [file Image_3.tif]
